# Investigating the relationship between older adults' basic psychological need satisfaction and daily positive emotional experience using experience sampling method: the multilevel mediation effect of life satisfaction

**DOI:** 10.1186/s41155-025-00366-x

**Published:** 2025-11-28

**Authors:** Ya-Ling Wang, Chih-Chi Liu

**Affiliations:** 1 Department of Adult and Continuing Education, National Taiwan Normal University, Taipei, Taiwan; 2 Department of Long-Term Care, Hsin Sheng Junior College of Medical Care and Management, No. 418, Sec. Gaoping, Zhongfeng Rd., Longtan Dist, Taoyuan, 325004 Taiwan

**Keywords:** Basic psychological need satisfaction, Life satisfaction, Emotional experience, Multilevel mediation modeling, Experience sampling method, Mobile application

## Abstract

**Background:**

The increasing proportion of older adults globally poses challenges to mental well-being, as aging is often accompanied by a decline in positive emotional experiences (PEE) and life satisfaction (LS). While previous studies have explored the relationship between basic psychological needs satisfaction (BPNS) and mental health in older adults, the role of life satisfaction as a mediating factor in the relationship between BPNS and daily emotional well-being remains underexplored.

**Objective:**

This study aims to investigate the relationship between BPNS and PEE among older adults and to examine the mediating role of LS. By employing the Experience Sampling Method (ESM) through a mobile application, the study provides real-time data on the emotional experiences of older adults.

**Methods:**

The study recruited 33 older adults (mean age = 63.24 years, SD = 5.92) who reported their daily emotional states through a mobile application three times a day for nine days. A total of 811 valid records were collected. Positive emotional experiences were assessed at the experience level, while BPNS and LS were assessed at the individual level. Data were analyzed using Hierarchical Linear Modeling (HLM) to examine the mediating effect of LS between BPNS and PEE.

**Results:**

Results revealed that LS significantly mediated the relationship between BPNS and PEE. Older adults with higher BPNS reported higher LS, which in turn enhanced their daily PEE. This finding supports the hypothesis that BPNS positively influences PEE through improved LS.

**Implications:**

The study highlights the importance of fostering BPNS to improve LS and daily positive emotions among older adults. The use of mobile applications to capture dynamic emotional states offers a practical and scalable approach for psychological research, particularly in aging populations. Further interventions could focus on enhancing BPNS to promote emotional well-being in older adults.

## Introduction

According to the United Nations (2020), the number of individuals aged 65 and over is projected to increase by more than 16% in 2050. While numerous studies have focused on the relationship between basic psychological needs satisfaction (BPNS) and the mental health of older adults (Liu & Wang, 2023; Tang et al., 2020; Wang & Hwang, 2020), it is important to note that a longer life expectancy does not necessarily equate to good mental health. In fact, older adults may experience a lower quality of life (Gómez-Bruton et al., 2021) or negative emotions (Wood & Joseph, 2010). Therefore, investigating the sources of daily emotions among older adults is imperative for enhancing their well-being. The aim of the present study is to identify factors that explain the sources of positive emotional experiences (PEE) among older adults.

The prevalence of mobile phones and the beneficial impact of digital technology on older adults has been well-documented in recent research (Marston et al., 2016; Xu et al., 2020), highlighting the necessity of digital technology and information products in daily life. For example, previous studies have encouraged individuals aged 85 and over to engage with technology products and participate in digital games (Marston et al., 2016). Moreover, a systematic review of the literature indicates that interventions involving video games have positive effects on the physical and mental health of older adults (Xu et al., 2020).

Previous studies have employed smartphone applications to collect data on individuals' daily mental health (Simons et al., 2020; Wang & Hwang, 2020). For example, Simons et al. (2020) discovered a positive correlation between feelings of abundance and emotions, as well as a negative correlation with negative emotions. In another study investigating the relationship between daily activities and basic psychological needs satisfaction, it was found that daily activities impact the psychological needs satisfaction of older adults (e.g., learning was positively correlated with autonomy needs satisfaction, but negatively correlated with competence needs satisfaction) (Wang & Hwang, 2020). Over time, older individuals experience changes in their mental health status, such as well-being, life satisfaction, emotional states, and psychological needs (Simons et al., 2020; Wang & Hwang, 2020). However, studies employing mobile phones and experience sampling methods (ESM) to understand the mental health of older adults in their daily lives are still uncommon. In order to address the existing research gap, the present study aims to develop a multilevel model that explores the theoretical predictors of older adults' daily emotional experiences and examines their associations with BPNS and LS.

### Precursor factors of positive emotional experiences

Previous studies have found that high levels of BPNS and LS are associated with greater well-being and positive emotions. Research has examined the theoretical antecedents and correlates of positive emotions (Businaro et al., 2015; Demir & Davidson, 2013; Gunnell et al., 2014). For instance, Gunnell et al. (2014) demonstrated that BPNS could be a crucial factor in promoting well-being (Gunnell et al., 2014). Similarly, Demir and Davidson (2013) suggested that satisfaction with BPNS was universally associated with psychological well-being and positive emotions. Moreover, well-being is regarded as an emotional state that is often expressed through LS (Bastian et al., 2014; Ryan & Deci, 2001), and research has shown that individuals with high LS tend to experience more positive emotions (Businaro et al., 2015). Therefore, this study considers BPNS and LS to be fundamental variables that may be theoretically related to PEE. Therefore, the possible associations between BPNS, LS, and PEE will be discussed below, and hypotheses for the current study will be proposed.

#### Basic psychological needs satisfaction (BPNS)

Studies in different contexts consistently reported that BPNS is positively related to positive emotions. BPNS is a part of the theories of Self-Determination Theory (SDT; Deci & Ryan, 2000; Ryan & Deci, 2000), which posits that three innate fundamental psychological needs (autonomy, competence, and relatedness) exist. Prior research has established that BPNS is linked to positive emotional experiences. For instance, Demir and Davidson (2013) found that BPNS was the strongest correlate of happiness in their cross-sectional analysis. In addition, other studies have investigated the relationship between BPNS and emotions in various contexts, such as teacher-student relationships (Klassen et al., 2012) and leisure sports (Teixeira et al., 2018), both of which have consistently reported that good BPNS is associated with positive emotions. Furthermore, a meta-analysis of the relationship between BPNS and positive and negative emotions also found that high positive emotions were significantly and positively associated with autonomy, competence, and relatedness (Stanley et al., 2021). With regard to the research on older adults, Liu and Wang (2023) found that older adults with different levels of BPNS experience different emotions when using information and communication technology. Specifically, the results of Liu and Wang’s study indicated that older adults with higher BPNS tend to have better emotional experiences. Based on the aforementioned findings, this study hypothesized that BPNS would be positively associated with PEE.

#### Life satisfaction (LS)

Higher levels of LS have been found to be associated with more positive emotional experiences, and this may also be true for older adults. LS, a subjective evaluation of one’s life, encompasses an individual's overall satisfaction and contentment with life (Volodina et al., 2019). For older adults, good LS is an indicator of successful aging (Mhaoláin et al., 2012). Extensive research has demonstrated that LS is related to emotional experiences (Bastian et al., 2014; Kuppens et al., 2008). For instance, Kuppens et al. (2008) found a positive correlation between LS and emotional experiences in countries where self-expression is open and free. Moreover, individuals with higher levels of LS tend to experience more positive emotions (Bastian et al., 2014). In addition, previous studies have indicated that positive emotional experiences in older adults may be closely related to their life satisfaction. For example, Celik et al. (2018) found that older adults’ feeling of sadness or melancholy was associated with lower life satisfaction. This suggests that older adults who are less satisfied with their lives may have less positive daily emotional experiences. These studies suggest that higher LS is associated with more positive emotional states. Our study supposed a positive relationship between LS and PEE.

### A multilevel mediation model approach: life satisfaction as the mechanism

Research had investigated the relationships between BPNS and LS (Volodina et al., 2019). For instance, it was suggested that BPNS was positively associated with LS and BPNS could also enhance LS (Volodina et al., 2019). Existing literature provides evidence that BPNS is a positive predictor of LS across different stages of life, and can be applied to the field of career and job research. For instance, Hollifield and Conger (2015) found that the BPNS of siblings was associated with their emerging adulthood LS in longitudinal analyses, and Butkovic et al. (2020) reported that adults and middle-aged adults with higher self-esteem and BPNS had higher LS. Moreover, Greguras and Diefendorff (2010) showed that BPNS is a predictor of employee LS, and Olčar et al. (2019) found that the satisfaction of psychological needs can enhance flow experience at work and lead to higher LS for primary school teachers. A systematic review and meta-analysis by Tang et al. (2020) also revealed a positive correlation between BPNS satisfaction and LS in older adults.

Overall, past studies have clarified the relationship between BPNS and LS among different study subjects and individuals. Based on the above findings, we hypothesized that LS would mediate the association between BPNS and PEE in a pattern consistent with theoretical expectations. Specifically, we proposed that older adults with higher levels of BPNS would report better LS, which would be associated with higher levels of PEE as reported through a mobile application on a daily basis.

### Theoretical rationale for directional hypotheses

Our directional hypotheses are grounded in Self-Determination Theory and the multilevel structure of our longitudinal data. SDT posits that psychological needs satisfaction serves as a fundamental antecedent to well-being outcomes (Ryan & Deci, 2000). In our study design, BPNS and LS were measured at the individual level as relatively stable characteristics, while positive emotional experiences were assessed repeatedly across nine days at the experience level.

The temporal stability of BPNS and LS as individual-level traits, compared to the more variable nature of daily emotional experiences measured through ESM, provides theoretical support for the proposed directional relationships. Previous longitudinal studies have demonstrated that BPNS measured at earlier time points predicts subsequent life satisfaction (Tang et al., 2021), and that stable individual differences in life satisfaction influence day-to-day emotional experiences over time. Our multilevel analysis tests whether individual-level characteristics (BPNS and LS) are associated with patterns of daily emotional experiences in ways consistent with these theoretical predictions.

### Conceptual framework

To ensure conceptual clarity and address potential concerns about construct overlap, we provide explicit definitions and distinctions between our key variables, particularly their classification as traits versus states and their temporal characteristics.

#### Basic psychological needs satisfaction (BPNS)—individual-level trait

BPNS represents a relatively stable individual difference characteristic reflecting the chronic degree to which an individual's fundamental psychological needs for autonomy (volition and self-direction), competence (effectiveness and mastery), and relatedness (connection and belonging) are satisfied across contexts and time (Ryan & Deci, 2000). While daily fluctuations in need satisfaction may occur, BPNS as measured in this study captures individuals' general, trait-like patterns of need fulfillment that remain relatively stable over weeks to months.

#### Life satisfaction (LS)—individual-level trait

Life satisfaction is conceptualized as a cognitive-evaluative component of subjective well-being, representing individuals' conscious, global judgments about their life as a whole (Diener et al., 1985). Unlike momentary emotions, life satisfaction reflects stable, trait-like evaluative assessments that integrate various life domains and experiences over extended periods. This construct captures the cognitive rather than affective aspects of well-being and demonstrates considerable temporal stability.

#### Positive emotional experience (PEE)—experience-level state

Positive emotional experiences represent momentary, state-like affective responses that fluctuate throughout daily life in response to situations, interactions, and internal processes (Russell, 2003). Unlike the relatively stable traits of BPNS and life satisfaction, positive emotions are characterized by their temporal variability, contextual sensitivity, and within-person fluctuation patterns.

#### Theoretical rationale for multilevel relationships

The distinction between trait-like characteristics (BPNS and LS) and state-like experiences (PEE) provides the theoretical foundation for our multilevel mediation model. Stable individual differences in psychological need satisfaction are theorized to influence global life evaluations (life satisfaction), which in turn shape the frequency and intensity of daily positive emotional experiences. This conceptualization aligns with hierarchical models of well-being that distinguish between stable dispositional factors and dynamic experiential outcomes (Schimmack, 2008).

### Design of the current study

The current study explores the intricate relationships among older adults’ basic psychological needs satisfaction (BPNS), life satisfaction (LS), and positive emotional experiences (PEE), with a strong emphasis on leveraging digital tools for psychological research. Recognizing the increasing digitalization of daily life, even among older populations, this study utilized the Experience Sampling Method (ESM) through the widely adopted mobile application, Line. This innovative approach enabled real-time, ecologically valid data collection three times a day over a nine-day period, offering a unique window into the participants’ momentary and daily emotional states.

By integrating ESM with a familiar and accessible mobile platform, this study not only captures dynamic emotional processes in older adults but also demonstrates the feasibility of using digital tools to enhance the efficiency of psychological research. Thirty-three older adults (mean age = 63.24 years, SD = 5.92; 81.8% female) participated in the study, generating 811 valid data points. Participants reported their daily PEE via the app, while individual-level measures of BPNS and LS were assessed separately. Hierarchical Linear Modeling (HLM) was employed to analyze the nested data, testing the hypothesis that LS mediates the relationship between BPNS and PEE. Specifically, we posited that higher BPNS leads to greater LS, which in turn promotes more frequent and intense daily PEE.

This study highlights the potential of mobile applications as a practical and scalable tool for conducting psychological research, particularly with aging populations. By bridging the gap between traditional survey methods and digital innovations, this work not only advances theoretical understanding of emotional well-being in older adults but also demonstrates how digital tools can facilitate engagement and participation in research. The findings offer actionable insights for developing digital interventions and policies aimed at enhancing psychological well-being among older populations, paving the way for future studies to further utilize digital methodologies in aging research.

### Contributions of the current study

While Self-Determination Theory provides a robust theoretical foundation for understanding the relationships between BPNS, life satisfaction, and well-being, several critical gaps remain in our understanding of how these relationships manifest in older adults' daily lives. First, existing research has predominantly relied on retrospective, cross-sectional methodologies that cannot capture the dynamic, real-time processes through which psychological needs satisfaction influences momentary emotional experiences in naturalistic settings. Second, the multilevel mechanisms through which stable individual characteristics (BPNS and life satisfaction) influence daily emotional fluctuations have not been empirically demonstrated using ecologically valid methods in aging populations. Third, the successful integration of familiar mobile technology for intensive longitudinal research with older adults remains underexplored, despite its potential to revolutionize data collection and intervention delivery in this population.

The current study addresses these gaps by: (1) providing the first real-time, ecologically valid examination of BPNS-emotion relationships in older adults' natural environments using experience sampling methodology; (2) demonstrating multilevel mediation mechanisms that bridge stable individual characteristics with moment-to-moment emotional experiences; (3) establishing the feasibility of using widely-adopted communication platforms (Line app) for high-compliance psychological research in aging populations; and (4) revealing the specific temporal and contextual conditions under which theoretical relationships manifest in daily life, moving beyond general theoretical predictions to actionable insights for interventions targeting psychological well-being in older adults.

## Method

### Participants and experience sampling method (ESM)

We recruited 33 older adults using purposive sampling through community senior centers and lifelong learning institutions in urban areas of Taiwan with specific inclusion criteria: (a) active Line application users, (b) willingness to participate in a nine-day intensive data collection period, and (c) demonstrated proficiency with mobile technology. Taiwan's cultural context, influenced by Confucian values emphasizing social harmony, family connections, and intergenerational relationships (Chuang & Wang, 2018; Yang, 2019), may be particularly relevant to understanding positive emotional experiences and psychological need satisfaction among older adults, as these cultural values often emphasize relational well-being and social connectedness (Wang et al., 2016). The age distribution of participants was: *M* = 63.24 years, *SD* = 5.92, range = 52–75 years, Mdn = 63 years, Q1 = 59 years, Q3 = 67 years. This distribution represents a diverse sample across different stages of older adulthood, enhancing the generalizability of our findings within the older adult population.

All participants were active users of the Line mobile application—87.5% had completed at least high school and all of the participants demonstrated sufficient proficiency with Line—thereby ensuring a high level of engagement and data quality. Over a 9-day period, each participant received three prompts per day via Line using signal-contingent sampling methodology, with one prompt delivered during each designated time period (morning, afternoon, and evening), and completed a total of 811 valid momentary records, in which they reported and rated their emotional status on a slider scale. This signal-contingent approach ensured systematic coverage of different periods of the day while maintaining the ecological validity characteristic of experience sampling methodology, allowing participants to report their emotional states in their natural environments when prompted by the system. The 80 missing data points (9.0% drop-out rate) were primarily attributed to participants not having their mobile phones available at the time of the prompt, as reported during post-study debriefing sessions. Most participants indicated that missing responses occurred without any particular reason other than not using their phone at the moment the prompt was received. Missing data are common and expected in experience sampling methodology (Schimmack, 2008; Scollon et al., 2003), as participants are instructed to respond only when convenient and when the prompt is noticed, in order to preserve ecological validity and minimize disruption to daily life. The sample was predominantly female (81.8%, *n* = 27), reflecting typical community-based participation patterns in Taiwan (e.g., Liu & Wang, 2023); although this gender imbalance may limit generalizability to older men, it accurately represents the demographic composition of active older-adult Line users. All participants provided written informed consent prior to data collection, and received a full debriefing upon study completion.

### Experience level measure

Positive emotional experience (PEE). To examine the participants’ current emotional state, participants were required to rate their emotional valence with the item “How do you feel right now? (1 = strongly upset, 4 = neutral, 7 = strongly happy)” as they received the reminder message.

### Individual level measures

Individual-level measures of BPNS and life satisfaction were administered at the beginning of the study, prior to the commencement of the 9-day experience sampling period. Participants completed these assessments during the initial orientation session, ensuring that their responses reflected their general psychological state rather than momentary fluctuations. This timing allowed us to examine how stable individual characteristics (measured at baseline) relate to patterns of daily emotional experiences (measured repeatedly over the 9-day period).

#### Basic psychological need satisfaction (BPNS)

The Basic Psychological Needs Satisfaction scale was based on the framework of the Basic Psychological Need Theory (Ryan, 1995; Ryan & Deci, 2000). The instrument consists of 21 items across three dimensions representing the three fundamental psychological needs, assessed on a 7-point Likert scale (1 = strongly disagree, 4 = neutral, 7 = strongly agree):

1. Autonomy (7 items): This dimension assesses the degree to which individuals feel volitional and self-directed in their actions. Sample items include: "I feel like I am free to decide for myself how to live my life," "I feel a sense of choice and freedom in the things I undertake," and "I feel my choices express my true self."
2. Competence (6 items): This dimension measures feelings of effectiveness and mastery in one's activities and environment. Sample items include: "I feel a sense of accomplishment most of the time," "I feel capable and effective in my actions," and "I feel I can successfully complete difficult tasks."
3. Relatedness (8 items): This dimension evaluates the sense of connection and belonging with others. Sample items include: "I really like the people I interact with," "I feel close and connected with other people who are important to me," and "I feel loved and cared about by those around me."

Consistent with Self-Determination Theory's conceptualization of these needs as interconnected and mutually reinforcing, we calculated the total BPNS score as the average of all 21 items, with higher scores indicating greater overall satisfaction of basic psychological needs.

#### Life satisfaction (LS)

The Chinese version instrument was translated by Wu and Yao (2006) based on the Satisfaction with Life Scale (SWLS; Diener et al., 1985). The instrument included five questions as LS and subjective well-being: (1) In most ways my life is close to my ideal; (2) The conditions of my life are excellent; (3) I am satisfied with my life; (4) So far I have gotten the important things I want in life; (5) If I could live my life over, I would change almost nothing (1 = strongly disagree, 4 = neutral, 7 = strongly agree).

### Data analysis

Due to the multi-level nature of our data structure, we analyzed the data using a hierarchical linear modeling (HLM) approach. The program and version used in this study is HLM8.0. The HLM approach allows us to account for the non-independence of experience-level measures from each individual (i.e., individuals’ LS and BPNS). The equation for the current study were as follows:

The Level 1 equation used to explain positive emotional experience (Y) is:

$$Y_{ij}=\beta_{0j}+\varepsilon_{ij}$$

The Level 2 equations are:

$$\beta_{0j}=\gamma_{00}+\gamma_{01\;}\left(\mathrm{BPNS}\right)+\gamma_{02\;}\left(\mathrm{LS}\right)+\mu_{0j}$$

Mixed model:

$$Y_{ij}=\gamma_{00}+\gamma_{01}\;\left(\mathrm{BPNS}\right)+\gamma_{02}\left(\mathrm{LS}\right)+\mu_{0j}+\varepsilon_{ij}$$

In addition, in order to examine our hypothesis of mediating effect, the current research also conducted regression analyses in which several regression analyses were conducted and the significance of the coefficients was examined in each step (Judd & Kenny, 1981).

## Results and discussions

### Descriptive statistics, correlation analysis and reliability

Table 1 contains means, standard deviations, correlation analysis and reliability for the major variables. From Table 1, it can be seen that there is a positive correlation between participants' satisfaction with basic psychological needs and their positive emotional experiences. It suggests that if participants' daily basic psychological needs are more satisfied, their emotional experiences may also be better, and vice versa Table 1.

**Table 1:** Descriptive statistics

Descriptive statistics			Correlation analysis	Reliability analysis	
	Mean	SD	LS	ICC (1)	ICC (2)
Level 1					
PEE	5.27	1.19		0.43	0.95
Level 2					
BPNS -Autonomy -Competence -Relatedness	5.48 5.41 5.59 5.45	0.66 0.68 0.60 0.70	.60^**^ .49^**^ .58^**^ .41^*^		
LS	5.25	1.10	-		

*PEE* positive emotional experience, *BPNS* basic psychological need satisfaction, *LS* life satisfaction

^*^p < .05

^**^p <.01

To verify whether the data in the current study meets the assumptions for conducting HLM, we first established a null model. The equations are:

$$Y_{ij}=\beta_{0j}+\varepsilon_{ij}$$

$$\beta_{0j}=\gamma_{00}+\mu_{0j}$$

Based on the aforementioned model, the reliability analysis yielded an Intraclass Correlation Coefficient (ICC) (1) value of 0.43 and ICC (2) value of 0.95. According to Myers (1972) recommendations, an ICC value should be above 0.25 to demonstrate the reliability of the data analyzed using a HLM approach. In addition, ICC (2) is used to assess the reliability of the overall mean (Glick & Roberts, 1984), and a threshold of above 0.7 is commonly employed as a criterion for judging reliability. Based on the results of the reliability analysis above, it is demonstrated that the current data is suitable for analysis using the HLM.

The current study leverages the visualized graphs in Fig. 2 to depict each participant’s daily average positive emotional experience (PEE) scores—computed from three prompts per day over a nine-day period. In this figure, the X-axis represents individual participants and the Y-axis their averaged PEE, with each line tracing a participant’s PEE trajectory. Although overlapping points (when multiple participants reported similar scores on the same days) can obscure certain data, the visualization nonetheless reveals substantial individual differences in both baseline PEE levels and daily fluctuation patterns. These observed variances across participants and days underscore the nested structure of the data and justify the use of hierarchical linear modeling (HLM) for proper analysis.

### Relationship between older adults’ BPNS and LS

All reported coefficients in the HLM analyses are unstandardized coefficients, allowing for direct comparison of effect sizes across different variables (see Table 2). The analysis predicting LS revealed the expected and significant effect of BPNS (*B* = 0.55, *p* <.001, *SD* = 0.24). The result indicated that older adults who have higher satisfaction with their BPNS showed significantly better LS. Additionally, correlation analyses (see Table 1) examining the relationships between LS and individual BPNS subdimensions showed that LS was significantly correlated with all three dimensions: Autonomy (*r* =.49, *p* <.01), Competence (*r* =.58, *p* <.01), and Relatedness (*r* =.41, *p* <.05). These findings suggest that while all three psychological needs contribute to life satisfaction, competence satisfaction showed the strongest association, followed by autonomy and relatedness satisfaction.

**Table 2:** Basic psychological need satisfaction and mediator predicting positive emotional experience

	PEE *B* (SD)		
Variables	Model A	Model B	Model C
BPNS	0.56^***^ (0.16)		0.21(0.18)
LS		0.45^***^ (0.10)	0.38^**^(0.12)

*BPNS* basic psychological need satisfaction, *LS* life satisfaction, *PEE* positive emotional experience

^**^p < *.01*

^***^p < *.001*

Our results of this study are consistent with those of previous studies (Neubauer et al., 2017; Tang et al., 2021). Well-being is often interpreted as a subjective assessment of a person's LS (Ryan & Deci, 2001). A cross-cultural study to understand the contribution of autonomous motivation and BPNS to well-being among elderly retired people from Chinese and French's result shows that psychological need satisfaction promotes their well-being (Tang et al., 2021). Also, research shows that the competence dimension (e.g., the perceptiveness of basic target activities) of BPNS, positively predicts the LS of older adults (Neubauer et al., 2017). Thus, BPNS is probably the most important predictor of LS. Older adults who have better BPNS will have better LS.

### Mediating effect of LS

**Fig. 1:**
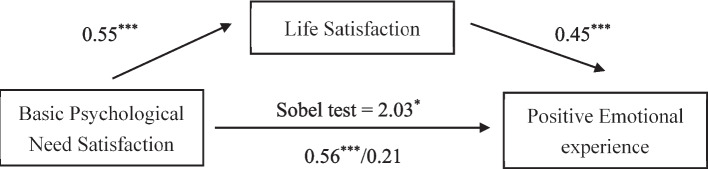
The mediation effect of life satisfaction between basic psychological need satisfaction and positive emotional experience

Results of the mediation effect of LS presented in Fig. 1 support the mediation effect. In addition, the coefficient for the direct relationship between BPNS and positive emotional experience decreased from a significant 0.56 (*p* <.001) to 0.21 (*ns*) after accounting for the effect of LS (*B* = 0.38, *p* <.01). The results supported our hypothetical model and were in line with self-determination theory (Deci & Ryan, 2000; Ryan & Deci, 2000), showing that satisfaction of BPNS was related to a positive PEE (Demir & Davidson, 2013; Klassen et al., 2012; Stanley et al., 2021; Teixeira et al., 2018), and better LS (Olčar et al., 2019).

**Fig. 2:**
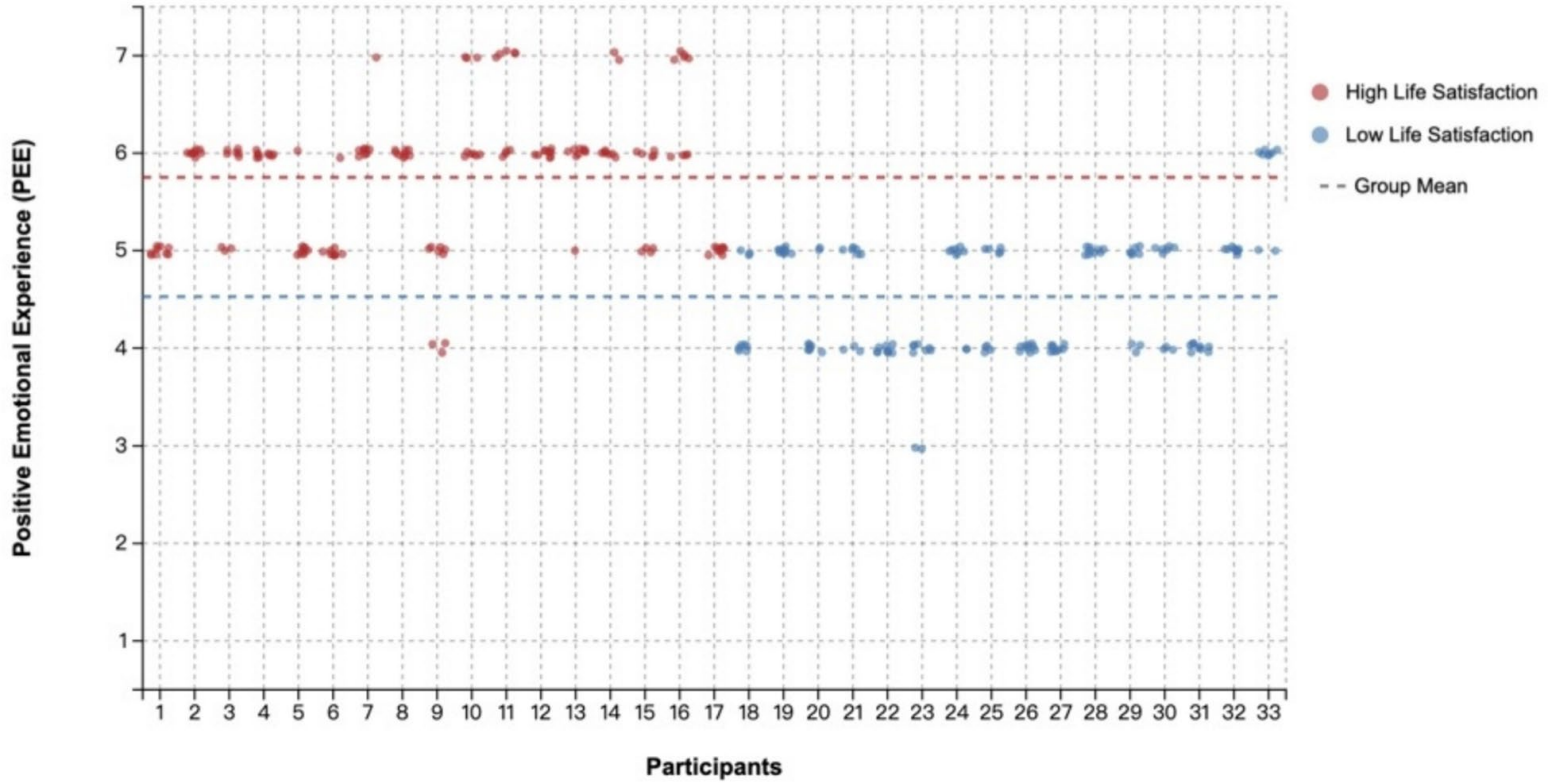
Distribution of positive emotional experiences among participants with different levels of life satisfaction. *Note:* Participants are ordered by LS scores from highest to lowest. Each line represents one participant's trajectory of positive emotional experiences (PEE) across the nine-day study period. Each point represents the PEE score for each participant (3 daily measurements per day). Lines connect the daily average PEE scores for each participant across the nine days. The Y-axis represents PEE scores ranging from 1 (strongly upset) to 7 (strongly happy). Due to overlapping data points when participants reported similar PEE scores on the same days, not all individual data points are visually distinguishable in the figure. The substantial between-person and within-person variability in PEE patterns justifies the use of hierarchical linear modeling to account for the nested data structure

Figure 2 presents the distribution of PEE for each participant in a visual manner, with participants categorized as high (above the mean) or low LS represented by different colors, to provide a clearer representation of the current distribution of the research data. In the visualization, the red portion represents participants with higher life satisfaction, as evidenced by their higher average daily PEE values, as seen in Fig. 2. Conversely, the blue portion represents participants with lower life satisfaction, characterized by lower average daily PEE. Such visualized results contribute to a more concise and clear interpretation of the findings in the current study.

Research evidence consistently demonstrates a positive correlation between BPNS and LS (Kloos et al., 2019). Additionally, higher levels of LS have been empirically linked to better PEE outcomes among individuals (Gueldner et al., 2001). A longitudinal study conducted in the Netherlands revealed that the BPNS of frail older adults may impact their LS. Specifically, the autonomy aspect of BPNS was found to be related to higher levels of LS, indicating that greater levels of BPNS can enhance LS (Kloos et al., 2019). In addition, LS and BPNS have been found to be closely related to PEE in older adults. Liu and Wang (2023)'s study both found that when the relatedness needs in the BPNS were satisfied, older adults had more positive emotional experiences when using information and communication technology (ICT). In addition, when older adults report higher levels of life satisfaction, they are more likely to have a positive outlook on their daily experiences and are less likely to experience negative emotions.

In summary, the cultivation of life satisfaction is critical for older individuals, as it contributes not only to positive emotions but also to successful aging (Mhaoláin et al., 2012). Therefore, greater attention and guidance should be provided to promote the development of life satisfaction in older adults. This could start with satisfying the BPNS of older adults, such as by enhancing their intergenerational support, which has been found to satisfy their need for relatedness in BPNS (Yang et al., 2022) and improve their life satisfaction (Chen & Jordan, 2018). Therefore, based on past research and current findings, intergenerational support may further improve the PEE of older adults by enhancing their BPNS and LS.

### Technology and smartphones are used as research tools for investigating the psychological status of older adults

Our study aimed to gather information on the mental health of older adults, specifically their BPNS, LS, and PEE, through the use of a mobile application. In today's society, computer-mediated communication has become an essential tool for gathering information. Furthermore, many recent studies have encouraged senior citizens to use digital video game products to maintain and promote their physical and mental health (Drazich et al., 2020; Xu et al., 2020). Adding to that, communication technologies have also been used to improve the well-being (Fang et al., 2018) and emotional states (Fang et al., 2019) of the elderly. However, most of these studies are cross-sectional or only focus on the results during the time when the particular product is used.

Further, mobile phone applications with ESM have been used in several studies to observe a wide range of conditions in everyday life (Liu & Wang, 2023; Simons et al., 2020; Wang & Hwang, 2020; Wouters et al., 2018). For example, a study examined the relationship between feelings of abundance and emotions in daily life among adults (Simons et al., 2020). However, the use of mobile applications with ESM to collect the psychological states of older adults is still relatively rare. Therefore, our study employed a mobile phone app with ESM to collect daily emotions from older adults (reference from Liu & Wang, 2023; Wang & Hwang, 2020), showcasing their ability and regularity in using smartphones. Finally, our research points to the fact that technology not only promotes mental health but also serves as a tool and method of investigation.

### Cultural context and generalizability

Our findings should be interpreted within the Taiwanese cultural context, which may influence both the manifestation and relationships of our key variables. The relatively high levels of life satisfaction observed in our sample may reflect cultural values that emphasize acceptance, family support, and finding meaning in later life stages, which are prominent in ethnic Chinese society (Cheng & Chan, 2006).

While Self-Determination Theory posits that basic psychological needs are universal, the ways in which these needs are satisfied may vary across cultures. In Taiwan, the relatedness need may be particularly influenced by collectivistic cultural values and strong family ties, while autonomy satisfaction may be expressed differently than in more individualistic cultures (Chen & Liu, 2019). Future cross-cultural research comparing these relationships across different cultural contexts would enhance our understanding of universal versus culture-specific aspects of psychological well-being in older adults.

The successful implementation of mobile technology (Line app) in our study also reflects Taiwan's advanced digital infrastructure and older adults' increasing technology adoption, which may not be generalizable to all cultural or economic contexts.

### Research highlights and innovative

Past research has indicated the need for further development in the use of ESM for elderly individuals (Myin-Germeys et al., 2018). The current study conducted in Taiwan is a pioneering effort in investigating the sources of older adults' PEE by utilizing a mobile Line application as an effective tool integrated with traditional ESM. The study's implications for future research and practical application are noteworthy as it adopts ESM to measure older adults' momentary emotional responses, a method that is widely used in previous studies to assess emotional states, including older adults’ situation (e.g., Carstensen et al., 2011). As to give our own unique contributions, what distinguishes this study from previous ones will be our innovative approach of using the most popular mobile communication application in Taiwan (i.e., Line) to conduct ESM for the assessment and evaluation of older adults' emotional processing and stability, rather than monitoring them in person, making phone calls, sending alerts by a smartphone program, or asking them to carry an electronic pager and a booklet every day. This research approach can also avoid the reluctance or operational errors that may occur when older adults use unfamiliar technology products as research tools. Based on the above, the current study suggests that future researchers should consider using such an approach (i.e., using communication applications to survey the elderly) to expand the progress of research tools and address potential problems encountered with past research tools (such as the need for elderly individuals to learn how to use new survey tools) to enhance research participation.

Furthermore, understanding emotional well-being and psychological flourishing in later life has become increasingly important as populations age globally. The current study primarily focuses on the PEE of older adults, which represents a critical component of emotional well-being that is inherently not a static phenomenon but rather fluctuates dynamically across daily experiences. By capturing these real-time emotional fluctuations, our research contributes to a deeper understanding of how psychological factors influence day-to-day emotional flourishing in aging populations. Therefore, it is evident from the results and visualizations of the current study that a multilevel analysis (specifically, the HLM analysis method employed) is an effective approach for researchers to better capture real-world phenomena of emotional well-being in older adults.

### Limitations

Although this study made a significant effort to investigate the precursor factors of older adults' positive emotional experience by proposing a multilevel mediation model, it is important to interpret the findings with caution. Therefore, we outline the limitations and corresponding future directions as follows. Firstly, there may be other mediators or moderators that can explain the relationships between BPNS and PEE. It is possible that the effect of BPNS on PEE may not exist or become weaker under certain conditions. Thus, it is crucial to explore these boundary conditions. Secondly, while this study utilized a longitudinal ESM design to investigate older adults' responses over nine days, several factors limit causal inference. Although we measured positive emotional experiences repeatedly over time, BPNS and life satisfaction were assessed at the individual level rather than being tracked over time. Additionally, the relatively short timeframe may not capture meaningful changes in these more stable constructs. Therefore, our mediation analysis should be interpreted as testing theoretical mediation patterns consistent with Self-Determination Theory rather than establishing definitive causal mechanisms. Future studies could incorporate experimental manipulations of psychological need satisfaction or longer-term longitudinal designs with repeated measures of all constructs to provide stronger evidence for causal relationships. In addition, the majority of participants in the current study were women (i.e., 6 men and 27 women), so the results should be interpreted with caution. Future studies should actively promote the participation of older men in relevant research in order to provide a more comprehensive interpretation of older adults with different demographic characteristics.

Additionally, our recruitment strategy through community centers and the requirement for Line application proficiency may have introduced selection bias toward older adults who are more socially active, technologically literate, and possibly healthier than the general older adult population. This sampling approach may limit the generalizability of our findings to older adults with different levels of social engagement, technology proficiency, or health status. Future research should employ more diverse recruitment strategies, including outreach to older adults in various settings (e.g., assisted living facilities, rural communities) and potentially offering technology support to include those with limited digital literacy. Finally, as discussed above, our study design required proficiency with communication applications, which limits our findings to older adults who are comfortable with such technology. This technological requirement, combined with our community-based recruitment approach, may have created a sample that is not representative of the broader older adult population. Future research should compare the relationship between BPNS and PEE across older adults with varying levels of technology proficiency and social engagement.

## Conclusion

Mobile applications and ESM methods provide convenient ways for communities or researchers to understand the psychological experiences of older adults in their daily lives. In conclusion, our study found that LS mediated the relationship between BPNS and PEE. Older adults with higher levels of BPNS are more likely to experience better LS, which to enhances their daily PEE.

## Data Availability

The raw data supporting the conclusions of this article will be made available by the corresponding author (Chih-Chi Liu; liucc1126@hsc.edu.tw), without undue reservation. The data are not publicly available due to restrictions their containing information that could compromise the privacy of research participants.

## Abbreviations

BPNS: Basic Psychological Needs Satisfaction
PEE: Positive Emotional Experiences
LS: Life Satisfaction
ESM: Experience Sampling Method
HLM: Hierarchical Linear Modeling
SD: Standard deviation

## References

[CR1] Bastian, B., Kuppens, P., De Roover, K., & Diener, E. (2014). Is valuing positive emotion associated with life satisfaction? *Emotion,**14*(4), 639–645. 10.1037/a0036466 24749643 10.1037/a0036466

[CR2] Businaro, N., Pons, F., & Albanese, O. (2015). Do intelligence, intensity of felt emotions and emotional regulation have an impact on life satisfaction? A Quali-quantitative study on subjective wellbeing with Italian children aged 8–11. *Child Indicators Research,**8*(2), 439–458. 10.1007/s12187-014-9250-x

[CR3] Butkovic, A., Tomas, J., Spanic, A. M., Vukasovic Hlupic, T., & Bratko, D. (2020). Emerging adults versus middle-aged adults: Do they differ in psychological needs, self-esteem and life satisfaction. *Journal Of Happiness Studies,**21*(3), 779–798. 10.1007/s10902-019-00106-w

[CR4] Carstensen, L. L., Turan, B., Scheibe, S., Ram, N., Ersner-Hershfield, H., Samanez-Larkin, G. R., Brooks, K. P., & Nesselroade, J. R. (2011). Emotional experience improves with age: Evidence based on over 10 years of experience sampling. *Psychology and Aging,**26*(1), 21–33. 10.1037/a0021285. [Research Support, N.I.H., Extramural]. 20973600 10.1037/a0021285 PMC3332527

[CR5] Celik, S. S., Celik, Y., Hikmet, N., & Khan, M. M. (2018). Factors affecting life satisfaction of older adults in Turkey. *The International Journal of Aging and Human Development,**87*(4), 392–414. 10.1177/0091415017740677h 29124946 10.1177/0091415017740677

[CR6] Chen, C., & Liu, G. H. (2019). Successful aging and life satisfaction among older adults in Taiwan. *Journal of Cross-Cultural Gerontology,**34*(2), 243–258. 10.1007/978-94-017-9331-5_11

[CR7] Chen, J., & Jordan, L. P. (2018). Intergenerational support and life satisfaction of young-, old- and oldest-old adults in China. *Aging & Mental Health,**22*(3), 412–420. 10.1080/13607863.2016.1261798 27918203 10.1080/13607863.2016.1261798

[CR8] Cheng, S. T., & Chan, A. C. M. (2006). Filial piety and psychological well-being in well older Chinese. *Journal of Gerontology: Psychological Sciences,**61B*(5), 262–269. 10.1093/geronb/61.5.P262 10.1093/geronb/61.5.p262 16960229

[CR9] Chuang, S., & Wang, G. G. (2018). Confucian philosophy and influence on perceived values and behavioural orientations by Taiwan’s millennials. *Human Resource Development International,**21*(4), 362–381. 10.1080/13678868.2018.1433393

[CR10] Deci, E. L., & Ryan, R. M. (2000). The “what” and “why” of goal pursuits: Human needs and the self-determination of behavior. *Psychological Inquiry,**11*(4), 227–268.

[CR11] Demir, M., & Davidson, I. (2013). Toward a better understanding of the relationship between friendship and happiness: Perceived responses to capitalization attempts, feelings of mattering, and satisfaction of basic psychological needs in same-sex best friendships as predictors of happiness. *Journal Of Happiness Studies,**14*(2), 525–550. 10.1007/s10902-012-9341-7

[CR12] Diener, E., Emmons, R. A., Larsen, R. J., & Griffin, S. (1985). The satisfaction with life scale. *Journal of Personality Assessment,**49*(1), 71–75. 10.1207/s15327752jpa4901_13 16367493 10.1207/s15327752jpa4901_13

[CR13] Drazich, B. F., LaFave, S., Crane, B. M., Szanton, S. L., Carlson, M. C., Budhathoki, C., & Taylor, J. L. (2020). Exergames and depressive symptoms in older adults: A systematic review. *Games for Health Journal,**9*(5), 339–345. 10.1089/g4h.2019.0165 32551982 10.1089/g4h.2019.0165

[CR14] Fang, Y., Chau, A. K., Wong, A., Fung, H. H., & Woo, J. (2018). Information and communicative technology use enhances psychological well-being of older adults: The roles of age, social connectedness, and frailty status. *Aging & Mental HealTh,**22*(11), 1516–1524. 10.1080/13607863.2017.1358354 28777010 10.1080/13607863.2017.1358354

[CR15] Fang, Y.-M., Chun, L., & Chu, B.-C. (2019). Older adults’ usability and emotional reactions toward text, diagram, image, and animation interfaces for displaying health information. *Applied Sciences,**9*(6), 1058. 10.3390/app9061058

[CR16] Glick, W. H., & Roberts, K. H. (1984). Hypothesized interdependence, assumed independence. *Academy of Management Review,**9*(4), 722–735. 10.5465/amr.1984.4277611

[CR17] Gómez-Bruton, A., López-Torres, O., Gómez-Cabello, A., Rodríguez-Gomez, I., Pérez-Gómez, J., Pedrero-Chamizo, R., Gusi, N., Ara, I., Casajús, J. A., & Gonzalez-Gross, M. (2021). How important is current physical fitness for future quality of life? Results from an 8-year longitudinal study on older adults. *Experimental Gerontology,**149*, Article 111301. 10.1016/j.exger.2021.111301 33737074 10.1016/j.exger.2021.111301

[CR18] Greguras, G. J., & Diefendorff, J. M. (2010). Why does proactive personality predict employee life satisfaction and work behaviors? A field investigation of the mediating role of the self-concordance model. *Personnel Psychology,**63*(3), 539–560. 10.1111/j.1744-6570.2010.01180.x

[CR19] Gueldner, S. H., Loeb, S., Morris, D., Penrod, J., Bramlett, M., Johnston, L., & Schlotzhauer, P. (2001). A comparison of life satisfaction and mood in nursing home residents and community-dwelling elders. *Archives Of Psychiatric Nursing,**15*(5), 232–240. 10.1053/apnu.2001.27020 11584352 10.1053/apnu.2001.27020

[CR20] Gunnell, K. E., Crocker, P. R., Mack, D. E., Wilson, P. M., & Zumbo, B. D. (2014). Goal contents, motivation, psychological need satisfaction, well-being and physical activity: A test of self-determination theory over 6 months. *Psychology of Sport and Exercise*, *15*(1), 19–29. 10.1016/j.psychsport.2013.08.005

[CR21] Hollifield, C. R., & Conger, K. J. (2015). The role of siblings and psychological needs in predicting life satisfaction during emerging adulthood. *Emerging Adulthood,**3*(3), 143–153. 10.1177/2167696814561544

[CR22] Judd, C. M., & Kenny, D. A. (1981). Process analysis: Estimating mediation in evaluation research. *Evaluation Research,**5*, 602–619.

[CR23] Klassen, R. M., Perry, N. E., & Frenzel, A. C. (2012). Teachers’ relatedness with students: An underemphasized component of teachers’ basic psychological needs. *Journal Of Educational Psychology,**104*(1), 150–165. 10.1037/a0026253

[CR24] Kloos, N., Trompetter, H. R., Bohlmeijer, E. T., & Westerhof, G. J. (2019). Longitudinal associations of autonomy, relatedness, and competence with the well-being of nursing home residents. *The Gerontologist,**59*(4), 635–643. 10.1093/geront/gny005 29529210 10.1093/geront/gny005

[CR25] Kuppens, P., Realo, A., & Diener, E. (2008). The role of positive and negative emotions in life satisfaction judgment across nations. *Journal of Personality and Social Psychology,**95*(1), 66–75. 10.1037/0022-3514.95.1.66 18605852 10.1037/0022-3514.95.1.66

[CR26] Liu, C.-C., & Wang, Y.-L. (2023). Does ICT usage have a positive or negative effect on Taiwanese older adults’ emotional experiences? The moderating role of basic psychological needs satisfaction. *Journal of Intelligence,**11*(3), Article 46. 10.3390/jintelligence11030046 36976139 10.3390/jintelligence11030046 PMC10058521

[CR27] Marston, H. R., Freeman, S., Bishop, K. A., & Beech, C. L. (2016). A scoping review of digital gaming research involving older adults aged 85 and older. *Games for Health Journal,**5*(3), 157–174. 10.1089/g4h.2015.0087 27096726 10.1089/g4h.2015.0087

[CR28] Mhaoláin, A. M. N., Gallagher, D., Connell, H. O., Chin, A., Bruce, I., Hamilton, F., Teehee, E., Coen, R., Coakley, D., & Cunningham, C. (2012). Subjective well-being amongst community-dwelling elders: What determines satisfaction with life? Findings from the Dublin Healthy Aging Study. *International Psychogeriatrics,**24*(2), 316–323. 10.1017/S1041610211001360 22189624 10.1017/S1041610211001360

[CR29] Myers, J. L. (1972). *Fundamentals in experimental design* (2nd ed.). Allyn & Bacon.

[CR30] Myin-Germeys, I., Kasanova, Z., Vaessen, T., Vachon, H., Kirtley, O., Viechtbauer, W., & Reininghaus, U. (2018). Experience sampling methodology in mental health research: New insights and technical developments. *World Psychiatry,**17*(2), 123–132. 10.1002/wps.20513 29856567 10.1002/wps.20513 PMC5980621

[CR31] Neubauer, A. B., Schilling, O. K., & Wahl, H.-W. (2017). What do we need at the end of life? Competence, but not autonomy, predicts intraindividual fluctuations in subjective well-being in very old age. *The Journals of Gerontology Series b: Psychological Sciences and Social Sciences,**72*(3), 425–435. 26447166 10.1093/geronb/gbv052

[CR32] Olčar, D., Rijavec, M., & Golub, T. L. (2019). Primary school teachers’ life satisfaction: The role of life goals, basic psychological needs and flow at work. *Current Psychology,**38*(2), 320–329. 10.1007/s12144-017-9611-y

[CR33] Russell, J. A. (2003). Core affect and the psychological construction of emotion. *Psychological Review,**110*(1), 145–172. 10.1037/0033-295X.110.1.145 12529060 10.1037/0033-295x.110.1.145

[CR34] Ryan, R. M. (1995). Psychological needs and the facilitation of integrative processes. *Journal of Personality,**63*(3), 397–427. 10.1111/j.1467-6494.1995.tb00501.x 7562360 10.1111/j.1467-6494.1995.tb00501.x

[CR35] Ryan, R. M., & Deci, E. L. (2000). Self-determination theory and the facilitation of intrinsic motivation, social development, and well-being. *American Psychologist,**55*(1), 68–78. 10.1037/0003-066X.55.1.68 11392867 10.1037//0003-066x.55.1.68

[CR36] Ryan, R. M., & Deci, E. L. (2001). On happiness and human potentials: A review of research on hedonic and eudaimonic well-being. *Annual Review of Psychology,**52*, 141. 10.1146/annurev.psych.52.1.141 11148302 10.1146/annurev.psych.52.1.141

[CR37] Schimmack, U. (2008). The structure of subjective well-being. In M. Eid & R. J. Larsen (Eds.), *The science of subjective well-being* (pp. 97–123). Guilford Press.

[CR38] Scollon, C. N., Kim-Prieto, C., & Diener, E. (2003). Experience sampling: Promises and pitfalls, strengths and weaknesses. *Journal of Happiness Studies,**4*(1), 5–34. 10.1023/A:1023605205115

[CR40] Simons, M., Lataster, J., Peeters, S., Reijnders, J., Janssens, M., & Jacobs, N. (2020). Sense of abundance is associated with momentary positive and negative affect: An experience sampling study of trait gratitude in daily life. *Journal of Happiness Studies,**21*(6), 2229–2236. 10.1007/s10902-019-00181-z

[CR41] Stanley, P. J., Schutte, N. S., & Phillips, W. J. (2021). A meta-analytic investigation of the relationship between basic psychological need satisfaction and affect. *Journal of Positive School Psychology,**5*(1), 1–16.

[CR42] Tang, M., Wang, D., & Guerrien, A. (2020). A systematic review and meta-analysis on basic psychological need satisfaction, motivation, and well-being in later life: Contributions of self-determination theory. *PsyCh Journal,**9*(1), 5–33. 10.1002/pchj.293 31177644 10.1002/pchj.293

[CR43] Tang, M., Wang, D., & Guerrien, A. (2021). The contribution of basic psychological need satisfaction to psychological well-being via autonomous motivation among older adults: A cross-cultural study in China and France. *Frontiers in Psychology*. 10.3389/fpsyg.2021.734461 34803814 10.3389/fpsyg.2021.734461 PMC8600240

[CR44] Teixeira, D. S., Silva, M. N., & Palmeira, A. L. (2018). How does frustration make you feel? A motivational analysis in exercise context. *Motivation and Emotion,**42*(3), 419–428. 10.1007/s11031-018-9690-6

[CR45] United Nations. (2020). *World population prospects 2019: Highlights*. https://www.un.org/development/desa/publications/world-population-prospects-2019-highlights.html

[CR46] Volodina, A., Lindner, C., & Retelsdorf, J. (2019). Personality traits and basic psychological need satisfaction: Their relationship to apprentices’ life satisfaction and their satisfaction with vocational education and training. *International Journal of Educational Research,**93*, 197–209. 10.1016/j.ijer.2018.11.003

[CR48] Wang, Y.-L., & Hwang, M.-Y. (2020). Daily activities and psychological need satisfaction of elderly adults: The experience sampling method. *Educational Gerontology,**46*(9), 551–562. 10.1080/03601277.2020.1786780

[CR49] Wood, A. M., & Joseph, S. (2010). The absence of positive psychological (eudemonic) well-being as a risk factor for depression: A ten year cohort study. *Journal of Affective Disorders,**122*(3), 213–217. 10.1016/j.jad.2009.06.032 19706357 10.1016/j.jad.2009.06.032

[CR50] Wu, C.-H., & Yao, G. (2006). Analysis of factorial invariance across gender in the Taiwan version of the Satisfaction with Life Scale. *Personality and Individual Differences,**40*(6), 1259–1268. 10.1016/j.paid.2005.11.012

[CR51] Xu, W., Liang, H.-N., Baghaei, N., Wu Berberich, B., & Yue, Y. (2020). Health benefits of digital videogames for the aging population: A systematic review. *Games for Health Journal,**9*(6), 389–404. 10.1089/g4h.2019.0130 32589482 10.1089/g4h.2019.0130

[CR52] Yang, J. C. C. (2019). The shaping of academic culture in higher education in Taiwan: Confucianism, historic legacy, and Western influences. In *Higher Education and Belief Systems in the Asia Pacific Region: Knowledge, Spirituality, Religion, and Structures of Faith* (pp. 15–25). Singapore: Springer Singapore.

[CR53] Yang, Y.-T., Yao, M., Yang, Y.-W., Ye, Q., & Lin, T. (2022). Relationships between children-related factors, basic psychological need satisfaction, and multiple happiness among urban empty-nesters in China: A structural equation modeling. *BMC Geriatrics,**22*(1), 925. 10.1186/s12877-022-03640-0 36457076 10.1186/s12877-022-03640-0 PMC9713964

